# Association between body composition and metabolic dysfunction-associated steatotic liver disease in Chinese general population: a cross-sectional study stratified by sex and obesity status

**DOI:** 10.3389/fpubh.2026.1921631

**Published:** 2026-09-17

**Authors:** Xiaxian Tan, Xiaomin Hou, Yuan Wen, Yuhua Huang, Shenghua Qin

**Affiliations:** Health Management Center, Guilin People’s Hospital, Guilin, China

**Keywords:** BMR, body composition analysis, FFM/FM ratio, MASLD, obesity status, WHR

## Abstract

**Background:**

Metabolic dysfunction-associated steatotic liver disease (MASLD) is a global health challenge with rising prevalence, yet the sex- and obesity-specific associations of body composition indices—particularly the fat-free mass to fat mass (FFM/FM) ratio—with MASLD remain incompletely characterized. This study aimed to investigate sex- and obesity-related differences in body composition associations with MASLD in a Chinese general population.

**Methods:**

This retrospective cross-sectional study enrolled 1,209 participants (562 males, 647 females) who underwent health check-ups at Guilin People’s Hospital between 2020 and 2022. Body composition was measured by bioelectrical impedance analysis (BIA), and MASLD was diagnosed by abdominal ultrasound using the 2023 consensus criteria. Logistic regression models assessed associations between body composition indices and MASLD, stratified by sex and obesity status. Interaction tests, restricted cubic splines (RCS), E-value calculations, and receiver operating characteristic (ROC) analyses were performed.

**Results:**

Overall MASLD prevalence was 28.3%, significantly higher in males (39.9%) than in females (18.2%), particularly among the obese group (57.4% vs. 41.2%) and non-obese group (16.9% vs. 7.7%). After adjusting for confounders, a lower FFM/FM ratio was significantly associated with higher MASLD prevalence in all subgroups except the non-obese stratum, in which the association was of similar magnitude but borderline non-significant. (males: OR = 0.47, 95% CI 0.26–0.83, *p* = 0.009; females: OR = 0.35, 95% CI 0.13–0.95, *p* = 0.039; obese: OR = 0.49, 95% CI 0.26–0.92, *p* = 0.028; non-obese: OR = 0.46, 95% CI 0.21–1.01, *p* = 0.051). Interaction tests were not significant (sex: *p* = 0.554; obesity: *p* = 0.917). RCS revealed a linear dose–response relationship (overall *p* = 0.021; nonlinear *p* = 0.289). *E*-values ranged from 2.21 to 2.77, indicating robustness to unmeasured confounding. Waist-to-hip ratio (WHR) was independently associated with MASLD only in females (OR = 1.13, 95% CI 1.04–1.22, *p* = 0.003), and the combined FFM/FM + WHR model achieved the highest AUC in females (0.822).

**Conclusion:**

FFM/FM ratio shows a universal protective effect against MASLD across sex and obesity strata, while WHR has sex-specific predictive value only in women. The linear dose–response relationship supports continuous monitoring of muscle-fat balance, even in normal-weight individuals. BIA-derived body composition analysis may assist in identifying high-risk individuals for MASLD in large-scale health check-ups.

## Introduction

Metabolic dysfunction-associated steatotic liver disease (MASLD, previously termed nonalcoholic fatty liver disease, NAFLD) is a chronic liver condition characterized by hepatic steatosis closely linked to metabolic syndrome (1). Approximately 30% of the global adult population is affected, with similarly high prevalence rates of 25–30% in Asian populations, and the burden continues to rise (2, 3). MASLD can progress to metabolic dysfunction-associated steatohepatitis (MASH), cirrhosis, and even hepatocellular carcinoma, and it significantly increases the risk of cardiovascular disease and type 2 diabetes (4, 5). Therefore, identifying modifiable risk factors is of great clinical importance for the early prevention and management of MASLD.

Traditional obesity indices such as body mass index (BMI) and waist circumference cannot differentiate between muscle and fat distribution, limiting their ability to accurately assess metabolic risk (6). Recently, body composition analysis—particularly the ratio of fat-free mass to fat mass—has gained attention. Multiple studies have reported that low muscle mass and high fat mass are independently associated with MASLD (7, 8). However, current evidence remains incomplete. On one hand, the integrated index of muscle and fat—the fat-free mass to fat mass (FFM/FM) ratio— has been understudied in the Chinese population (9), and whether sex or obesity status modifies its association with MASLD is unclear (10). On the other hand, the dose–response relationship (linear vs. threshold) between FFM/FM and MASLD has not been established (11), and sex-specific predictive performance of body composition indices has not been compared.

Bioelectrical impedance analysis (BIA) is a technique for estimating the distribution and proportion of muscle and fat in human body by measuring electrical resistance. It has the advantages of being easy to use, non-invasive, time-saving, and radiation-free, and has a high correlation with other obesity assessment methods (such as DXA, CT, and MRI) (12–14). In this study, BIA was used to obtain FFM, FM, waist-to-hip ratio (WHR), and basal metabolic rate (BMR). MASLD was diagnosed by abdominal ultrasound; although liver biopsy remains the gold standard, ultrasound is widely accepted as a screening tool in large populations (15). Moreover, this study strictly follows the 2023 international consensus statement by adopting the new MASLD definition—hepatic steatosis plus at least one cardiometabolic risk factor—thus aligning the diagnosis more closely with metabolic dysfunction (1).

This study aimed to systematically evaluate the associations of body composition indices (particularly FFM/FM ratio and WHR) with MASLD prevalence in a Chinese general population, stratified by sex and obesity status. To address previous methodological gaps, we performed formal interaction tests for effect modification, used restricted cubic splines (RCS) to examine the dose–response relationship, calculated E-values to quantify the robustness to unmeasured confounding, and compared the area under the curve (AUC) of different indices for predicting MASLD by sex. We tested the hypotheses that the FFM/FM-MASLD association is linear and that sex modifies the predictive value of WHR.

## Materials and methods

### Study population

This retrospective cross-sectional study gathered clinical data from individuals who received health check-ups at the Health Management Center of Guilin People’s Hospital between January 2020 and December 2022. The following inclusion criteria were used to select participants: (1) Age ≥18 years old; (2)Underwent standard abdominal ultrasound, body composition analysis, and serum biochemistry; (3) No history of excessive alcohol consumption—defined as <140 g/week for men and <70 g/week for women according to the 2007 Asian Pacific Association for the Study of the Liver (APASL) guideline (16); (4) Absence of hepatic steatosis due to viruses, drugs, or autoimmune diseases; (5) Availability of all variables required for MASLD diagnosis, including body mass index (BMI), blood pressure, fasting blood glucose (FBG), triglycerides (TG), high-density lipoprotein cholesterol (HDL), low-density lipoprotein cholesterol (LDL), total cholesterol (TC), alanine aminotransferase (ALT), aspartate aminotransferase (AST), and gamma-glutamyl transferase (GGT). Exclusion criteria: (1) Patients with hepatic steatosis due to viral liver disease (positive for hepatitis B surface antigen or hepatitis C virus antibody), autoimmune liver disease, Wilson’s disease, drug-induced liver disease, or other causes; (2) Incomplete or unanalyzable clinical/biochemical data. (3) Excessive alcohol consumption (Alcohol consumption ≥140 g/week for men and ≥70 g/week for women). Figure 1 illustrates the screening process, culminating in the inclusion of 1,209 participants for further analysis.

**Figure 1:**
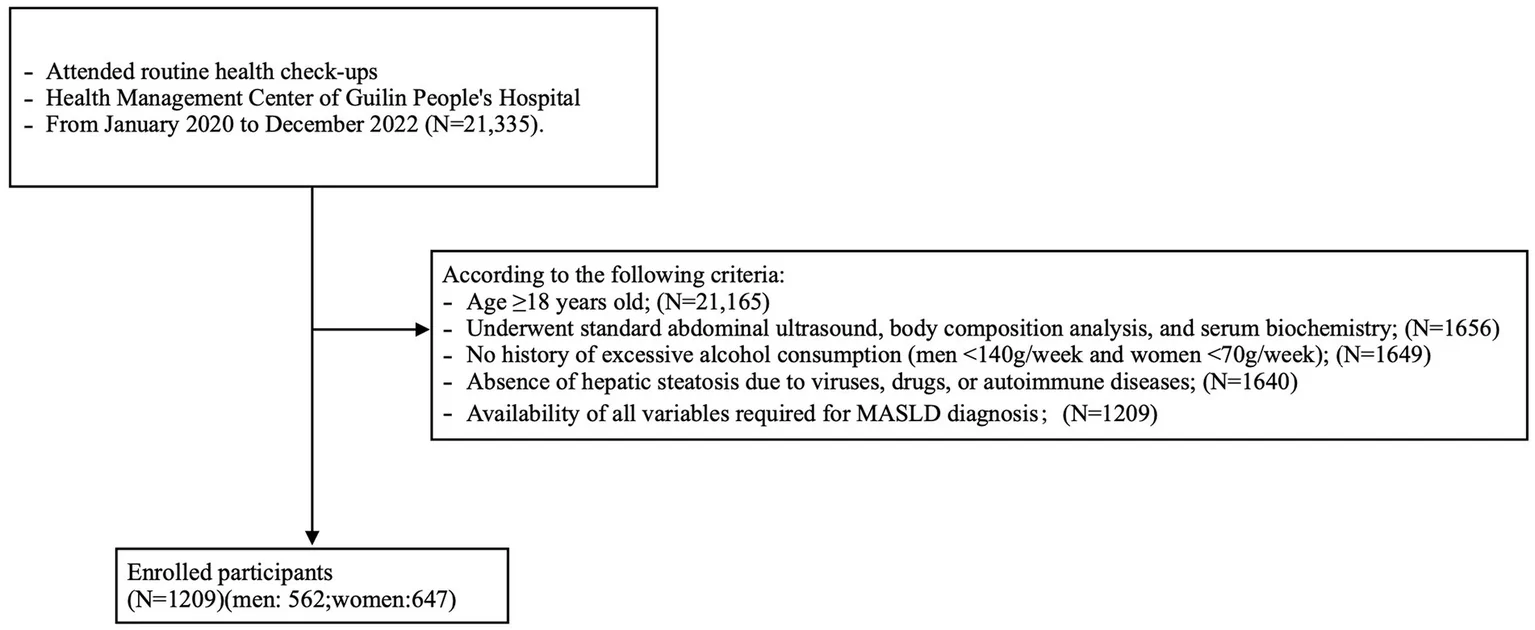
Enrollment flowchart of participants.

### Data collection

All participants were instructed to fast for at least 8 h before blood collection. Peripheral venous blood samples were drawn in the early morning using standard venipuncture technique into 5 mL vacuum tubes containing a clotting activator (serum separator tubes). Blood specimens were allowed to clot at room temperature for 30 min and then centrifuged at 3000 rpm for 10 min at 4 °C to obtain serum. Serum biochemical parameters, including TC, HDL, LDL, TG, FBG, AST, ALT, GGT, creatinine (Cr), blood urea nitrogen (BUN), and uric acid (UA), were measured using a fully automated clinical chemistry analyzer (cobas 8,000 c 702 module; Roche Diagnostics GmbH, Mannheim, Germany) with commercially available reagent kits (Roche Diagnostics, Mannheim, Germany). All assays were performed according to the International Federation of Clinical Chemistry and Laboratory Medicine (IFCC) reference procedures and the manufacturer’s instructions. Clinical laboratory quality control samples were run in each batch to ensure accuracy and reproducibility of the results. Demographic and clinical data, including age, sex, height, weight, body mass index, blood pressure, and heart rate, were extracted from the hospital’s electronic medical record system.

### Ultrasound examination

An experienced sonographer, unaware of the body composition data, conducted and interpreted the liver ultrasound. Fatty liver was diagnosed using a high-resolution B-mode ultrasound system with a 3.5 MHz probe (GE LOGIO F8 Expert, China) based on four imaging diagnostic criteria: liver parenchyma brightness, liver-kidney echo contrast, blood vessel blurring, and deep-wave velocity attenuation (17, 18).

### Bioelectrical impedance analysis

We measured height and weight according to standard procedures. According to the instruction manual, body composition was measured using BIA with the InBody570 (InBody, Seoul, Korea). All participants were barefoot, without metal jewelry, and fasted, completing the test in a quiet environment at approximately 25 °C. Body composition analysis provided measurements for FFM, FM, body fat percentage (BFP), BMR, and WHR. Examinations were performed by uniformly trained and qualified medical personnel and were blinded to the clinical information. The InBody570 system determines BMR by applying the formula: BMR = 21.6 × FFM (kg) + 370 (19, 20).

### Definitions

Given the prevalence of visceral fat accumulation in Asian populations, we considered BMI ≥ 25 kg/m^2^ as the threshold for diagnosing obesity (21). Thus, non-obese MASLD was defined as: individuals with a BMI < 25 kg/m^2^ and meeting diagnostic criteria for MASLD. Hypertension was determined by a systolic blood pressure (SBP) ≥ 140 mmHg and/or a diastolic blood pressure (DBP) ≥ 90 mmHg, or the use of antihypertensive drugs. The derived indicators are normalized according to the patient’s height, and the corresponding formula is as follows:


$$\mathrm{BMI}=\text{weight}\;(\mathrm{kg})/\text{heigh}t^{2}\;(m^{2})$$

$$\text{FFMI}=\mathrm{FFM}\;(\mathrm{kg})/\text{heigh}t^{2}\;(m^{2})$$

$$\mathrm{FMI}=\mathrm{FM}\;(\mathrm{kg})/\text{heigh}t^{2}\;(m^{2})$$

### Statistical analysis

Statistical analyses were conducted using SPSS version 27.0 (IBM Corp., Armonk, NY, United States) and R version 4.2 (R Foundation for Statistical Computing, Vienna, Austria). Normally distributed variables are reported as mean ± SD, while non-normally distributed variables are reported as median (P25-P75). The Kolmogorov–Smirnov test was used to assess normality. For group comparisons, continuous variables were analyzed using the independent *t*-test for normally distributed data and the Wilcoxon rank-sum test for non-normally distributed data; categorical variables were compared using the χ^2^ test. Univariate and multivariate logistic regression models were used to estimate the associations between body composition indices and MASLD prevalence. Odds ratios (OR) with 95% confidence intervals (CI) were reported. Three nested multivariable models were constructed with stepwise adjustment for potential confounders, selected *a priori* based on established literature and clinical plausibility: Model 0 was unadjusted (crude model). Model 1 was adjusted for age, sex (or BMI group, depending on stratification), hypertension, and heart rate. Model 2 was further adjusted for metabolic parameters that are recognized risk factors for MASLD, including FBG, UA, TG, HDL, and LDL. All tests were two-sided, and a *p*-value <0.05 was considered statistically significant. Effect modification was tested by including product terms (FFM/FM × sex and FFM/FM × obesity) in the total-sample logistic regression, using Wald test. Restricted cubic splines (4 knots at 5th, 35th, 65th, 95th percentiles) examined non-linearity (rms package). E-values were calculated (EValue package, rare = FALSE) to quantify robustness to unmeasured confounding. ROC curves stratified by sex and obesity status assessed predictive performance of WHR alone and WHR combined with FFM/FM; AUCs were compared by DeLong test. A forest plot (forestplot package) presented subgroup ORs (Model 2) with interaction *p*-values. An additional forest plot summarized the univariate associations of core body composition indices across subgroups. With 342 MASLD cases and 12 independent variables in Model 2, the events-per-variable (EPV = 28.5) exceeded the recommended minimum of 10, indicating adequate model stability. Formal statistical power was not calculated *a priori* for subgroup analyses. For the stratified analyses, the EPV for each subgroup was: male 18.7 (224/12), female 9.8 (118/12), obese 22.3 (267/12), and non-obese 6.3 (75/12), suggesting model stability was adequate for most subgroups, except the non-obese (EPV = 6.3), where the limited sample size may have reduced precision. No data were missing. Collinearity was ruled out (VIF < 5). Given the exploratory nature of the subgroup analyses, no adjustment for multiple comparisons was applied. These subgroup findings should therefore be interpreted as hypothesis-generating and warrant confirmation in future studies. Decision curve analysis (DCA) was performed using a validated manual calculation approach to evaluate the clinical utility of the combined WHR + FFM/FM model relative to WHR alone. The analysis was conducted across a clinically plausible range of threshold probabilities (0–0.5) in both the overall population and sex-stratified subgroups (Supplementary Figures 4 and 5).

## Results

### Basic characteristics of the enrolled participants

A total of 1,209 participants were enrolled, with 562 males (46.5%) and 647 females (53.5%). The mean age was 49.0 ± 12.5 years for males and 49.4 ± 11.7 years for females. The average BMI was 25.5 ± 3.4 kg/m^2^ for males and 23.6 ± 3.4 kg/m^2^ for females. The overall prevalence of MASLD was 28.3%, with significantly higher rates in males than in females (39.9% vs. 18.2%). This male predominance was consistent across both in the obese (57.4% vs. 41.2%) and non-obese (16.9% vs. 7.7%) subgroups. Interaction tests showed that the product terms FFM/FM × sex (*p* = 0.554) and FFM/FM × obesity status (*p* = 0.917) were not statistically significant, indicating that the strength of the association between FFM/FM ratio and MASLD did not differ significantly by sex or obesity status. Table 1 presents the clinical characteristics of participants categorized by sex.

**Table 1:** Clinical characteristics of participants stratified by sex.

Variables	Male (*n* = 562)			Female (*n* = 647)			MASLD (Male vs. Female)
	Non-MASLD (*n* = 338)	MASLD (*n* = 224)	*P*1	Non- MASLD (*n* = 529)	MASLD (*n* = 118)	*P*2	*P*3
Age (year)	50.20 ± 13.09	47.30 ± 11.40	0.007	48.26 ± 11.35	54.44 ± 12.19	<0.001	<0.001
BMI (kg/m^2^)	24.21 ± 2.9	27.52 ± 3.06	<0.001	22.85 ± 2.93	26.92 ± 3.64	<0.001	0.107
HBP	77(23.30%)	67(29.90%)	0.058	93(18.10%)	40(33.60%)	<0.001	0.450
SBP (mmHg)	125.82 ± 19.15	130.95 ± 18.29	0.001	121.53 ± 19.47	130.50 ± 20.84	<0.001	0.841
DBP (mmHg)	75.34 ± 12.86	80.09 ± 12.69	<0.001	70.52 ± 11.42	75.53 ± 12.09	<0.001	0.001
HR (beats/min)	75.04 ± 10.79	78.38 ± 10.94	<0.001	78.17 ± 10.83	79.30 ± 10.58	0.287	0.457
WHR	0.91 ± 0.05	0.96 ± 0.05	<0.001	0.88 ± 0.04	0.94 ± 0.05	<0.001	<0.001
BMR (kcal)	1465.41 ± 135.3	1554.71 ± 138.56	<0.001	1178.41 ± 95.85	1243.36 ± 107.22	<0.001	<0.001
FFM (kg)	50.71 ± 6.26	54.85 ± 6.41	<0.001	37.52 ± 3.88	40.43 ± 4.96	<0.001	<0.001
FM (kg)	16.80 ± 5.13	23.43 ± 6.45	<0.001	18.05 ± 5.04	25.01 ± 6.67	<0.001	0.034
BFP (%)	24.51 ± 5.35	29.68 ± 5.13	<0.001	32.03 ± 5.73	37.78 ± 4.95	<0.001	<0.001
FFMI (kg/m^2^)	18.15 ± 1.57	19.23 ± 1.48	<0.001	15.37 ± 1.19	16.58 ± 1.35	<0.001	<0.001
FMI (kg/m^2^)	6.04 ± 1.86	8.22 ± 2.18	<0.001	7.44 ± 2.17	10.29 ± 2.68	<0.001	<0.001
FFM/FM	3.03 (2.60–3.77)	2.43 (2.05–2.82)	<0.001	2.10 (1.78–2.54)	1.69 (1.47–1.90)	<0.001	<0.001
ALT(U/L)	18 (14–24)	29.50 (21–47)	<0.001	13 (10–18)	20 (15–25)	<0.001	<0.001
AST(U/L)	20 (18–24)	23 (19–29)	0.001	18 (15–21.80)	20 (17–23)	0.006	0.024
GGT (U/L)	28.50 (19–45)	49.50 (35–72)	<0.001	16 (12–22)	23 (18–36)	0.001	<0.001
BUN (mmol/L)	5.06 ± 1.30	4.95 ± 1.15	0.309	4.51 ± 1.20	4.77 ± 1.30	0.035	0.185
Cr (μmol/L)	82.80 (73–91)	81.50 (73–90)	0.17	59 (54–64)	61 (56–66.75)	0.031	<0.001
UA (μmol/L)	378.63 ± 77.57	433.35 ± 80.52	<0.001	286.18 ± 60.33	333.76 ± 82	<0.001	<0.001
FBG (mmol/L)	5.03 (4.67–5.36)	5.22 (4.80–5.75)	0.001	4.94 (4.56–5.24)	5.22 (4.81–5.76)	<0.001	0.833
TC (mmol/L)	4.82 (4.28–5.49)	5.26 (4.71–5.84)	<0.001	4.91 (4.35–5.44)	5.08 (4.31–5.61)	0.175	0.018
TG (mmol/L)	1.44 (0.99–1.98)	2.28 (1.74–3.16)	<0.001	1.06 (0.80–1.45)	1.69 (1.11–2.12)	<0.001	<0.001
LDL (mmol/L)	3.03 ± 0.78	3.44 ± 0.86	<0.001	2.83 ± 0.75	3.16 ± 0.82	<0.001	0.003
HDL (mmol/L)	1.25 ± 0.30	1.09 ± 0.31	<0.001	1.51 ± 0.32	1.28 ± 0.25	<0.001	<0.001

Normally distributed variables are presented as mean ± SD and skewed variables as median (P25-P75); group comparisons were performed using the *t*-test or Wilcoxon rank-sum test as appropriate. Categorical variables are presented as frequencies (percentages) and compared using the χ^2^ test. BMI, body mass index; HBP, high blood pressure; SBP, systolic blood pressure; DBP, diastolic blood pressure; HR, heart rate; WHR, Waist-to-hip ratio; BMR, basal metabolic rate; FFM, fat-free mass; FM, fat mass; PBF, percent body fat; FFMI, fat-free mass index; FMI, fat mass index; FFM/FM, fat-free mass to fat mass ratio; ALT, alanine aminotransferase; AST, aspartate aminotransferase; GGT, gamma-glutamyl transferase; BUN, blood urea nitrogen; Cr, creatinine; UA, uric acid; FBG, fasting blood glucose; TC, total cholesterol; TG, triglyceride; LDL, low-density lipoprotein; HDL, high-density lipoprotein.

### Characteristics of the participants with and without MASLD stratified by sex and BMI

Table 1 and Table 2 indicate that individuals with MASLD exhibited significantly higher levels of BMI, blood pressure, WHR, BMR, FFM, FM, BFP, FFMI, FMI, AST, ALT, GGT, UA, FBG, TG, and LDL, and significantly lower FFM/FM ratio and HDL, compared to those without MASLD, irrespective of sex or obesity status. As shown in Table 1, WHR, BMR, FFM, FFMI, FFM/FM, Cr, UA, TC, TG, and LDL were significantly higher in male MASLD participants than in female MASLD participants, whereas BFP, FMI, and HDL were significantly higher in female MASLD participants. Notably, male participants with MASLD were younger than those without MASLD (47.3 ± 11.40 vs. 50.2 ± 13.09, *p* < 0.05), while the opposite was true for females (54.44 ± 12.19 vs. 48.26 ± 11.35, *p* < 0.05), and male participants had a significantly younger age at MASLD diagnosis than females. In addition, non-obese male MASLD participants were similar in age to their obese counterparts, whereas non-obese female MASLD participants were older than obese ones, but this difference was not statistically significant (Schedule 2, Schedule 3).

**Table 2:** Clinical characteristics of participants stratified by BMI.

Variables	BMI < 25 kg/m^2^		BMI ≥ 25 kg/m^2^		*P*1	*P*2	*P3*
	Non-MASLD (*n* = 611)	MASLD (*n* = 75)	Non-MASLD (*n* = 256)	MASLD (*n* = 267)			
Male	202 (33.06%)	41 (54.67%)	136 (53.13%)	183 (68.54%)	<0.001	0.026	<0.001
Age (year)	48.32 ± 12.06	52.03 ± 11.10	50.68 ± 12.01	49.13 ± 12.37	0.012	0.068	0.148
BMI (kg/m^2^)	21.91 ± 2	23.50 ± 1.28	26.90 ± 1.78	28.39 ± 2.84	<0.001	<0.001	<0.001
HBP	104 (17.21%)	19 (25.33%)	66 (25.78%)	88 (32.96%)	0.077	0.208	0.072
SBP (mmHg)	121.43 ± 19.31	126.61 ± 19.70	127.66 ± 18.16	131.97 ± 18.91	0.029	0.032	0.008
DBP (mmHg)	71.27 ± 11.91	74.63 ± 11.42	75.29 ± 11.94	79.61 ± 12.79	0.021	0.002	<0.001
HR (beats/min)	77.05 ± 10.64	76.83 ± 10.62	76.81 ± 10.89	79.22 ± 10.82	0.868	0.09	0.011
WHR	0.88 ± 0.04	0.91 ± 0.04	0.93 ± 0.04	0.96 ± 0.05	<0.001	<0.001	<0.001
BMR (kcal)	1247.32 ± 153.51	1320.63 ± 158.29	1392.88 ± 196.13	1482.87 ± 191.19	<0.001	<0.001	<0.001
FFM (kg)	40.69 ± 6.78	44.01 ± 7.33	47.36 ± 9.08	51.52 ± 8.85	<0.001	<0.001	<0.001
FM (kg)	15.45 ± 3.78	17.78 ± 2.98	22.6 ± 4.29	25.71 ± 6.24	<0.001	<0.001	<0.001
BFP (%)	27.59 ± 6.1	29.07 ± 5.58	32.69 ± 6.63	33.43 ± 6.25	0.046	<0.001	0.193
FFMI (kg/m^2^)	15.79 ± 1.52	16.62 ± 1.41	18.04 ± 1.84	18.80 ± 1.76	<0.001	<0.001	<0.001
FMI (kg/m^2^)	6.09 ± 1.63	6.83 ± 1.43	8.81 ± 2.07	9.53 ± 2.49	<0.001	<0.001	<0.001
FFM/FM	2.58 (2.09–3.29)	2.53 (1.93–2.94)	2.02 (1.64–2.69)	2.04 (1.71–2.47)	0.005	<0.001	0.092
ALT(U/L)	14 (11–20)	22 (17–33)	17 (13–24)	26 (19–39)	<0.001	0.614	<0.001
AST(U/L)	18 (16–22)	22 (18–25.50)	20 (17–23)	21.80 (18–28)	<0.001	0.322	<0.001
GGT (U/L)	17 (13–28)	33 (20–54.50)	24.50 (18–40)	41 (26–63.50)	<0.001	0.233	<0.001
BUN (mmol/L)	4.64 ± 1.25	4.86 ± 1.46	4.92 ± 1.3	4.9 ± 1.13	0.148	0.837	0.807
Cr (μmol/L)	63 (56–76)	70 (59.50–84)	72 (59.85–85.25)	75 (64.50–86)	0.003	0.299	0.063
UA (μmol/L)	308.57 ± 77.72	379.83 ± 99.53	354.8 ± 80.23	404.37 ± 91.58	<0.001	0.045	<0.001
FBG (mmol/L)	4.93 (4.56–5.24)	5.09 (4.83–5.72)	5.09 (4.74–5.39)	5.25 (4.80–5.76)	<0.001	0.036	0.001
TC (mmol/L)	4.87 (4.31–5.40)	5.12 (4.50–5.64)	4.94 (4.38–5.56)	5.23 (4.59–5.78)	0.053	0.177	0.001
TG (mmol/L)	1.07 (0.81–1.60)	1.91 (1.38–2.63)	1.39 (1.08–1.95)	2.06 (1.62–2.88)	<0.001	0.781	<0.001
LDL (mmol/L)	2.85 ± 0.74	3.17 ± 0.8	3.07 ± 0.77	3.39 ± 0.87	<0.001	0.047	<0.001
HDL (mmol/L)	1.46 ± 0.33	1.17 ± 0.28	1.28 ± 0.31	1.15 ± 0.31	<0.001	0.667	<0.001

Normally distributed variables are presented as mean ± SD and skewed variables as median (P25-P75); group comparisons were performed using the t-test or Wilcoxon rank-sum test as appropriate. Categorical variables are presented as frequencies (percentages) and compared using the χ^2^ test. P1: MASLD group vs. NON- MASLD group among participants with BMI <25 kg/m^2^; P2: BMI <25 kg/m^2^ MASLD group versus BMI ≥ 25 kg/m^2^ MASLD group; P3: MASLD group vs. NON- MASLD group among participants with BMI ≥25 kg/m^2^. BMI, body mass index; HBP, high blood pressure; SBP, systolic blood pressure; DBP, diastolic blood pressure; HR, heart rate; WHR, Waist-to-hip ratio; BMR, basal metabolic rate; FFM, fat-free mass; FM, fat mass; PBF, percent body fat; FFMI, fat-free mass index; FMI, fat mass index; FFM/FM, fat-free mass to fat mass ratio; ALT, alanine aminotransferase; AST, aspartate aminotransferase; GGT, gamma-glutamyl transferase; BUN, blood urea nitrogen; Cr, creatinine; UA, uric acid; FBG, fasting blood glucose; TC, total cholesterol; TG, triglyceride; LDL, low-density lipoprotein; HDL, high-density lipoprotein.

Table 2 summarizes clinical characteristics stratified by BMI. Obese MASLD participants had significantly higher levels of BMI, blood pressure, WHR, BMR, FFM, FM, BFP, FFMI, and FMI compared to non-obese MASLD participants, whereas non-obese MASLD participants had a significantly higher FFM/FM ratio. Serum biochemistry showed that obese MASLD participants had significantly higher average levels of UA, FBG, and LDL than non-obese MASLD participants, but no significant differences in ALT, AST, GGT, Cr, TG, or HDL were observed between the two groups.

### Univariate analysis of MASLD prevalence stratified by sex and BMI

We performed univariate logistic regression analyses stratified by sex and BMI to assess the direction and strength of the association of each variable with MASLD. As shown in Table 3 and Supplementary Figure 1, BMI, WHR, BMR, FFM, FM, FFMI, FMI, UA, TG, FBG, LDL, and HDL were significantly associated with MASLD independent of sex and obesity status (*p* < 0.05). Except for obese MASLD participants, FFM/FM and BFP were significantly associated with MASLD in males, females, and non-obese participants. Additionally, hypertension, BUN, and Cr were significantly associated with MASLD in females (*p* < 0.05), but not in males. In obese MASLD participants, age, hypertension, BFP, FFM/FM, BUN, and Cr levels were not associated with MASLD prevalence, whereas in non-obese MASLD participants, hypertension, heart rate, and BUN were not associated with MASLD prevalence. TC was identified as a factor associated with MASLD, showing a significant association specifically in males and obese participants, but not in females or non-obese participants (female: OR, 1.17; *p* = 0.175; non-obese MASLD: OR, 1.29; *p* = 0.054).

**Table 3:** Univariate analysis of MASLD prevalence in different sex and obesity status stratified by sex and BMI.

	Male (*n* = 562)		Female (*n* = 647)		BMI < 25 kg/m^2^ (*n* = 686)		BMI ≥ 25 kg/m^2^ (*n* = 523)	
	OR (95% CI)	P1	OR (95% CI)	P2	OR (95% CI)	P3	OR (95% CI)	P4
Sex					0.41 (0.25–0.67)	<0.001	0.52 (0.36–0.74)	<0.001
Age	0.98 (0.97–1.00)	0.008	1.05 (1.03–1.07)	<0.001	1.03 (1.01–1.05)	0.012	0.99 (0.98–1.00)	0.148
BMI	1.49 (1.38–1.61)	<0.001	1.48 (1.36–1.60)	<0.001	1.78 (1.47–2.14)	<0.001	1.36 (1.24–1.49)	<0.001
HBP	1.45 (0.99–2.12)	0.059	2.40 (1.55–3.74)	<0.001	1.65 (0.94–2.90)	0.079	1.42 (0.97–2.07)	0.072
SBP	1.02 (1.01–1.02)	0.002	1.02 (1.01–1.03)	<0.001	1.01 (1.00–1.03)	0.03	1.01 (1.00–1.02)	0.009
DBP	1.03 (1.02–1.04)	<0.001	1.04 (1.02–1.05)	<0.001	1.02 (1.00–1.04)	0.022	1.03 (1.01–1.04)	<0.001
HR	1.03 (1.01–1.05)	0.001	1.01 (0.99–1.03)	0.287	1.00 (0.98–1.02)	0.868	1.02 (1.01–1.04)	0.012
WHR	1.23 (1.18–1.28)	<0.001	1.30 (1.24–1.38)	<0.001	1.23 (1.15–1.31)	<0.001	1.19 (1.14–1.24)	<0.001
BMR	1.60 (1.40–1.84)	<0.001	2.08 (1.66–2.61)	<0.001	1.34 (1.16–1.56)	<0.001	1.27 (1.16–1.39)	<0.001
FFM	1.11 (1.08–1.14)	<0.001	1.17 (1.12–1.23)	<0.001	1.07 (1.03–1.10)	<0.001	1.05 (1.03–1.07)	<0.001
FM	1.24 (1.19–1.30)	<0.001	1.24 (1.19–1.30)	<0.001	1.20 (1.12–1.29)	<0.001	1.13 (1.09–1.17)	<0.001
BFP	1.22 (1.17–1.27)	<0.001	1.21 (1.16–1.27)	<0.001	1.04 (1.00–1.09)	0.047	1.02 (0.99–1.05)	0.193
FFMI	1.58 (1.40–1.79)	<0.001	2.19 (1.81–2.64)	<0.001	1.43 (1.22–1.67)	<0.001	1.26 (1.14–1.40)	<0.001
FMI	1.79 (1.60–2.01)	<0.001	1.63 (1.46–1.80)	<0.001	1.35 (1.15–1.57)	<0.001	1.15 (1.06–1.25)	0.001
FFM/FM	0.25 (0.18–0.34)	<0.001	0.09 (0.05–0.16)	<0.001	0.73 (0.55–0.98)	0.034	0.79 (0.61–1.04)	0.092
ALT	1.04 (1.03–1.05)	<0.001	1.03 (1.02–1.05)	<0.001	1.03 (1.01–1.04)	<0.001	1.05 (1.04–1.07)	<0.001
AST	1.03 (1.01–1.05)	0.001	1.04 (1.02–1.06)	<0.001	1.04 (1.02–1.06)	<0.001	1.04 (1.02–1.07)	<0.001
GGT	1.01 (1.00–1.01)	<0.001	1.01 (1.01–1.02)	0.001	1.01 (1.00–1.02)	<0.001	1.01 (1.01–1.02)	<0.001
BUN	0.93 (0.81–1.07)	0.32	1.18 (1.01–1.38)	0.037	1.14 (0.95–1.36)	0.148	0.98 (0.85–1.13)	0.806
Cr	0.99 (0.98–1.00)	0.173	1.03 (1.01–1.04)	0.007	1.02 (1.01–1.03)	0.005	1.01 (1.00–1.02)	0.063
UA	1.09 (1.07–1.12)	<0.001	1.11 (1.07–1.14)	<0.001	1.09 (1.06–1.12)	<0.001	1.07 (1.05–1.09)	<0.001
FBG	1.26 (1.09–1.45)	0.002	1.43 (1.21–1.70)	<0.001	1.31 (1.16–1.49)	<0.001	1.38 (1.13–1.67)	0.002
TC	1.58 (1.32–1.91)	<0.001	1.17 (0.93–1.46)	0.175	1.29 (1.00–1.68)	0.054	1.37 (1.14–1.65)	0.001
TG	2.06 (1.71–2.49)	<0.001	2.39 (1.84–3.09)	<0.001	2.21 (1.74–2.80)	<0.001	1.85 (1.51–2.25)	<0.001
LDL	1.90 (1.51–2.40)	<0.001	1.70 (1.31–2.20)	<0.001	1.72 (1.26–2.35)	0.001	1.65 (1.32–2.07)	<0.001
HDL	0.13 (0.06–0.25)	<0.001	0.06 (0.02–0.13)	<0.001	0.04 (0.02–0.10)	<0.001	0.23 (0.12–0.43)	<0.001

*p*-values were calculated from the univariate logistic regression models. BMI, body mass index; HBP, high blood pressure; SBP, systolic blood pressure; DBP, diastolic blood pressure; HR, heart rate; WHR, Waist-to-hip ratio; BMR, basal metabolic rate; FFM, fat-free mass; FM, fat mass; PBF, percent body fat; FFMI, fat-free mass index; FMI, fat mass index; FFM/FM, fat-free mass to fat mass ratio; ALT, alanine aminotransferase; AST, aspartate aminotransferase; GGT, gamma-glutamyl transferase; BUN, blood urea nitrogen; Cr, creatinine; UA, uric acid; FBG, fasting blood glucose; TC, total cholesterol; TG, triglyceride; LDL, low-density lipoprotein; HDL, high-density lipoprotein.

RCS analysis revealed a significant overall association between the FFM/FM ratio and MASLD prevalence (overall test: χ^2^ = 9.71, df = 3, *p* = 0.021), while the non-linear component was not statistically significant (χ^2^ = 2.49, df = 2, *p* = 0.289), indicating a linear dose–response relationship. Supplementary Figure 2 presents the RCS plot for the overall population, showing a consistent linear inverse association between FFM/FM ratio and MASLD prevalence. Subgroup-specific RCS plots were not generated due to limited sample sizes in certain strata (particularly non-obese, n = 75), which precluded stable model estimation.

### Sex-stratified multivariate analysis of body composition and MASLD prevalence

We performed sex-stratified multivariate logistic regression analyses to assess the independent associations of body composition parameters with MASLD prevalence and to explore potential sex differences. Table 4 and Figure 2 show that after adjusting for age, BMI, blood pressure, and heart rate, the FFM/FM ratio was significantly inversely associated with MASLD prevalence, while WHR and BMR showed positive associations, regardless of sex. After further adjustment for FBG, TG, UA, HDL, and LDL, FFM/FM remained significantly negatively associated with MASLD prevalence in both sexes (male: OR, 0.47; 95% CI, 0.26–0.83; *p* = 0.009; female: OR, 0.35; 95% CI, 0.13–0.95; *p* = 0.039). Notably, only in females did WHR and BMR show a positive association with MASLD prevalence (WHR: OR, 1.13; 95% CI, 1.04–1.22; *p* = 0.003; BMR: OR, 2.02; 95% CI, 1.40–2.91; *p* < 0.001). In males, although positive associations were observed, they did not reach statistical significance (WHR: OR, 1.06; 95% CI, 0.99–1.14; *p* = 0.076; BMR: OR, 1.21; 95% CI, 0.97–1.52; *p* = 0.096).

**Table 4:** Multivariate analysis of MASLD prevalence in different sex and obesity status stratified by sex and BMI.

Subgroup	Model 0		Model 1		Model 2		E-value
	OR (95% CI)	*p*	OR (95% CI)	*p*	OR (95% CI)	*p*	
Male (*n* = 562)							
FFM/FM	0.34 (0.22–0.53)	<0.001	0.45 (0.27–0.76)	0.002	0.47 (0.26–0.83)	0.009	2.28
WHR	1.07 (1.01–1.14)	0.022	1.09 (1.02–1.16)	0.008	1.06 (0.99–1.14)	0.076	
BMR	1.50 (1.27–1.76)	<0.001	1.30 (1.04–1.58)	0.019	1.21 (0.97–1.52)	0.096	
Female (*n* = 647)							
FFM/FM	0.20 (0.09–0.43)	<0.001	0.25 (0.10–0.66)	0.005	0.35 (0.13–0.95)	0.039	2.77
WHR	1.15 (1.07–1.23)	<0.001	1.11 (1.04–1.20)	0.004	1.13 (1.04–1.22)	0.003	
BMR	1.69 (1.27–2.24)	<0.001	2.18 (1.55–3.08)	<0.001	2.02 (1.40–2.91)	<0.001	
BMI≥25 kg/m^2^ (*n* = 686)							
FFM/FM	0.47 (0.27–0.81)	0.007	0.46 (0.25–0.84)	0.011	0.49 (0.26–0.92)	0.028	2.21
WHR	1.12 (1.06–1.19)	<0.001	1.12 (1.05–1.18)	<0.001	1.10 (1.03–1.16)	0.003	
BMR	1.44 (1.21–1.71)	<0.001	1.50 (1.21–1.86)	<0.001	1.42 (1.13–1.77)	0.002	
BMI<25 kg/m^2^ (*n* = 523)							
FFM/FM	0.50 (0.27–0.92)	0.025	0.41 (0.21–0.80)	0.009	0.46 (0.21–1.01)	0.051	-
WHR	1.13 (1.04–1.23)	0.003	1.10 (1.01–1.20)	0.032	1.10 (1.00–1.21)	0.049	
BMR	1.52 (1.14–2.03)	0.004	1.47 (1.05–2.06)	0.027	1.33 (0.89–1.97)	0.161	

*p*-values were calculated from the multivariate logistic regression models. Model 0 was crude model. Model 1 adjusted for age, sex (or BMI_group), hypertension, and heart rate. Model 2 was further adjusted for FBG, UA, TG, HDL, and LDL based on model 1. WHR, Waist-to-hip ratio; BMR, basal metabolic rate. *E*-value calculated using the *E*-Value package in R with rare = FALSE (common outcome). Based on the point estimate of the odds ratio. –, Not calculated due to non-significance.

**Figure 2:**
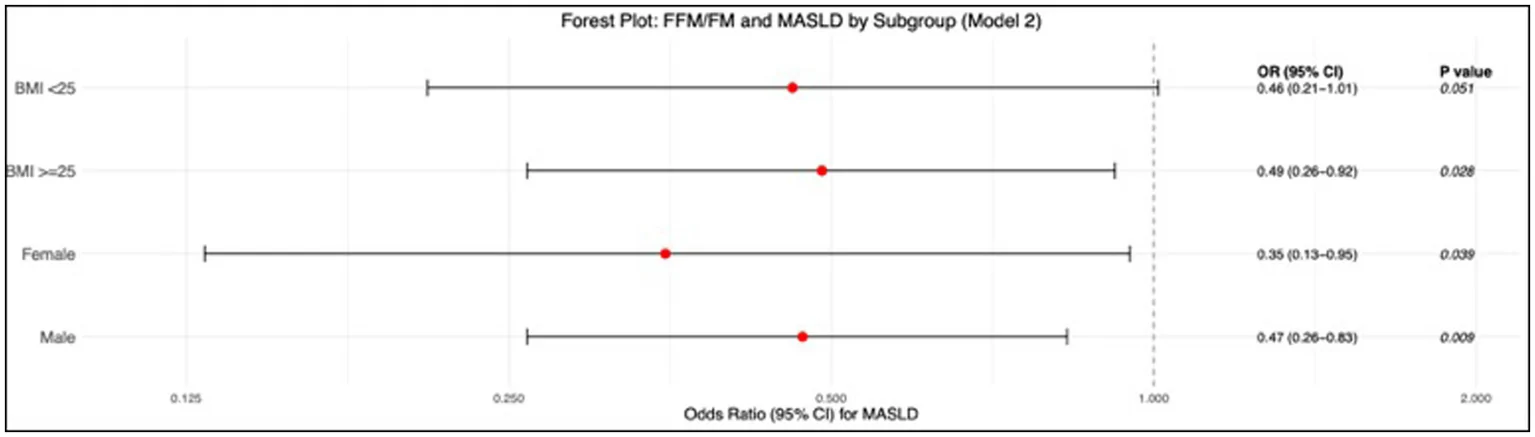
Forest plot of multivariate logistic regression results.

Additionally, we evaluated the area under the curve (AUC) for predicting MASLD using FFM/FM alone, WHR alone, and their combination, stratified by sex. As shown in Table 5 and Figure 3, in males, the AUC of FFM/FM was 0.756 (95% CI, 0.72–0.80), WHR was 0.76 (95% CI, 0.72–0.80), and the combined model was 0.78 (95%CI, 0.74–0.82). The combined model had a significantly higher AUC than FFM/FM alone (*p* = 0.023) and WHR alone (*p* = 0.024). In females, the AUC of FFM/FM was 0.78 (95% CI, 0.73–0.82), WHR was 0.81 (95% CI,0.77–0.85), and the combined model was 0.82 (95% CI, 0.783–0.861). The combined model had a significantly higher AUC than FFM/FM alone (*p* = 0.001), but the improvement over WHR alone was not statistically significant (*p* = 0.07). Figure 3 displays the corresponding ROC curves, illustrating the incremental predictive value of adding FFM/FM to WHR, particularly in males. Schedule 4 and Supplementary Figure 3 shows ROC analyses stratified by obesity status. Adding FFM/FM to WHR did not significantly improve AUC in either the obese (0.703 to 0.706, *p* = 0.381) or non-obese (0.741 to 0.740, *p* = 0.845) subgroups.

**Table 5:** AUC comparison for predicting MASLD by sex.

Sex	Predictor	AUC (95% CI)	*p* (vs. Combined)
Male (*n* = 562)	Combined	0.778 (0.740–0.816)	—
	WHR	0.762 (0.723–0.801)	0.024
	FFM/FM	0.756 (0.717–0.796)	0.023
Female (*n* = 647)	Combined	0.822 (0.783–0.861)	—
	WHR	0.808 (0.766–0.850)	0.07
	FFM/FM	0.776 (0.734–0.818)	0.001

*p*-values from DeLong test for correlated ROC curves.

**Figure 3:**
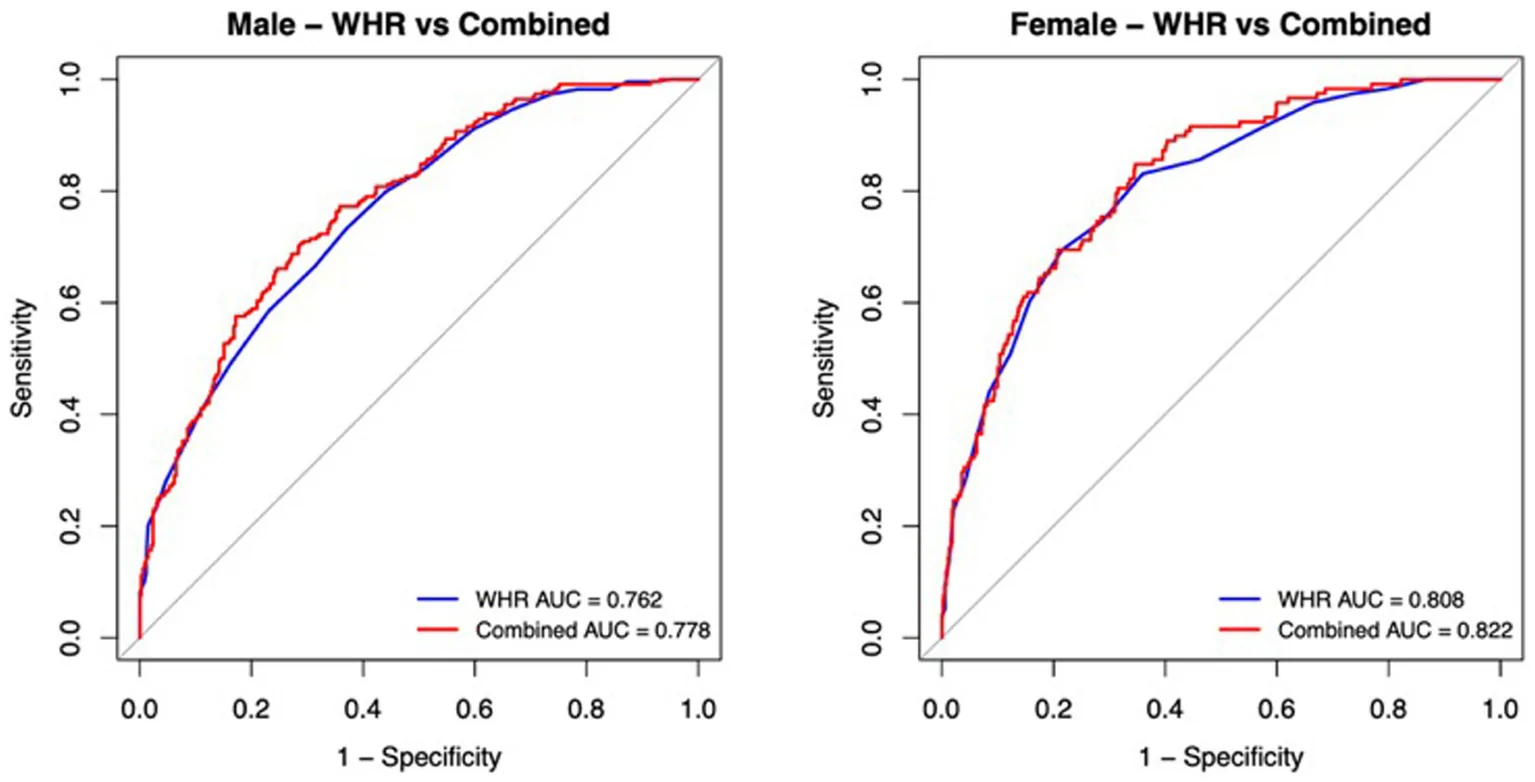
Sex‑stratified ROC curves for WHR, and combined FFM/FM model in predicting MASLD.

### Obesity-stratified multivariate analysis of body composition and MASLD prevalence

We performed BMI-stratified multivariate logistic regression analyses to assess the independent associations of body composition parameters with MASLD prevalence in non-obese and obese participants. As shown in Table 4 and Figure 2, after adjustment for age, sex, blood pressure, and heart rate, the FFM/FM ratio was significantly inversely associated with MASLD prevalence, while WHR and BMR showed positive associations, independent of obesity status. After further adjustment for FBG, TG, UA, HDL, and LDL, the FFM/FM ratio remained significantly negatively associated with MASLD prevalence in obese participants; a similar inverse association was observed in non-obese participants but did not reach statistical significance (obesity: OR, 0.49; 95% CI, 0.26–0.92; *p* = 0.028; non-obesity: OR, 0.46; 95% CI, 0.21–1.01; *p* = 0.051). WHR was significantly positively associated with MASLD prevalence in both groups (obesity NAFLD: OR, 1.10; 95% CI, 1.03–1.16; *p* = 0.003; non-obesity MASLD: OR, 1.10; 95% CI, 1.00–1.21; *p* = 0.049). However, BMR was significantly positively associated with MASLD prevalence only in obese participants (OR, 1.42; 95% CI, 1.13–1.77; *p* = 0.002); A positive association was observed in non-obese participants but did not reach significance (OR, 1.33; 95% CI, 0.89–1.97; *p* = 0.161).

Figure 2 presents a forest plot summarizing the multivariable-adjusted ORs for FFM/FM ratio across all sex and obesity subgroups. The plot shows that the inverse association between FFM/FM ratio and MASLD was consistent across all subgroups, with ORs ranging from 0.35 (females) to 0.49 (obese participants). All subgroups except the non-obese group showed statistically significant associations, with the non-obese subgroup exhibiting a consistent trend (OR = 0.46; 95% CI: 0.21–1.01; *p* = 0.051).

In supplementary analyses, we compared the predictive performance of the combined WHR + FFM/FM model with two established non-invasive indices for MASLD, the triglyceride-glucose (TyG) index and the hepatic steatosis index (HSI), across sex and obesity subgroups (Schedule 5). In females, the combined model (AUC = 0.822) significantly outperformed TyG (AUC = 0.762, *p* = 0.046) and showed comparable performance to HSI (*p* = 0.347). In males, the combined model was comparable to TyG (AUC: 0.778 vs. 0.775, *p* = 0.923) but inferior to HSI (AUC: 0.778 vs. 0.837, *p* = 0.002). In obesity-stratified analyses, TyG and HSI showed higher AUCs than the combined model in non-obese individuals, while all three indices performed modestly in the obese subgroup.

DCA showed that adding FFM/FM to WHR did not increase net benefit in either the overall population or sex-stratified subgroups (Supplementary Figures 4 and 5).

### Sensitivity analysis

To assess the robustness of our findings to potential unmeasured confounding, we calculated *E*-values using the EValue package in R with rare = FALSE to account for the non-rare outcome (overall MASLD prevalence >15%). *E*-values derived from the point estimates of Model 2 were 2.28 for males, 2.77 for females, and 2.21 for obese participants, indicating moderate robustness to unmeasured confounding. *E*-values based on the 95% CI lower bounds were not estimable due to the common-outcome transformation (rare = FALSE). For the non-obese subgroup, the 95% CI crossed the null (OR = 0.46, 95% CI: 0.21–1.01), and thus the E-value was not computed.

## Discussion

In this study, we systematically evaluated the associations between body composition indices and MASLD prevalence in a Chinese general population, stratified by sex and obesity status. We found that the FFM/FM ratio was independently and robustly negatively associated with MASLD, and this association was consistent across male, female, obese, and non-obese subgroups. Interaction tests did not reach statistical significance, indicating that the protective effect of the FFM/FM ratio was not modified by sex or obesity status. Moreover, this is the first study to demonstrate a linear dose–response relationship between the FFM/FM ratio and MASLD in a Chinese population using RCS analysis, suggesting that any improvement in the FFM/FM ratio may confer proportional metabolic benefits. In contrast, WHR was independently associated with MASLD only in female.

Beyond confirming the association, our study provides novel evidence on the shape of the dose–response relationship. Previous studies have mostly focused on whether the FFM/FM ratio or similar integrated indices are associated with MASLD, whereas our study is the first to clarify the linear dose–response relationship. This finding has important implications. First, a lower FFM/FM ratio correlates with a proportionally higher risk of MASLD. Second, clinically, it implies that no protective “threshold” exists, and intervention efforts should aim for continuous improvement of the FFM/FM ratio. Studies by Dai et al. (22) and Xu et al. (9) have already confirmed the general importance of muscle-fat balance in NAFLD risk stratification. Building on that evidence, our study used RCS to validate the linear shape of the association, providing more precise quantitative evidence for a dose-dependent protective effect of muscle-fat balance in MASLD pathogenesis.

The consistent inverse association of FFM/FM with MASLD across subgroups prompted us to further explore sex-specific patterns. Our sex-stratified analyses provide new insights into the sexual dimorphism of MASLD. Consistent with previous reports, we observed a higher prevalence of NAFLD and an earlier age of onset in males than in females (10, 23). However, a more noteworthy finding is that, despite the lower prevalence in females, affected women exhibited a significantly lower FFM/FM ratio than affected men, and the OR for the FFM/FM ratio in multivariable regression was lower in females than in males, suggesting that a low FFM/FM ratio may have a relatively greater impact on MASLD risk in females. Sex hormones underlie this biological difference: estrogen modulates glucose and lipid metabolism via the ERα signaling pathway, exerts anti-inflammatory and antioxidant effects, and protects premenopausal women from liver damage (24, 25). Nevertheless, the protective effect of estrogen does not fully eliminate the risk attributable to muscle loss. In males, lower estrogen levels favor the accumulation of visceral adipose tissue, which is metabolically more harmful, leading to earlier and more prevalent NAFLD (26, 27). Visceral adipose tissue secretes more inflammatory cytokines, participates in insulin resistance, and is more strongly associated with NAFLD and adverse metabolic outcomes than subcutaneous fat (8, 28). Combined with sex differences in body composition (higher FFM in males, higher FM in females), these findings strongly suggest that the sex difference in the FFM/FM ratio is not merely a quantitative difference but reflects a qualitative divergence in how muscle-fat imbalance drives MASLD between sexes.

In addition to the sex differences, we observed a clinically important trend in the non-obese subgroup that warrants discussion. Regarding the non-obese subgroup, we observed a clinically important trend: although the protective association of the FFM/FM ratio did not reach statistical significance, the direction and magnitude of the effect were highly consistent with those in other subgroups. This borderline non-significance likely reflects limited statistical power due to the small number of MASLD cases in this subgroup (*n* = 75) rather than a true absence of biological effect. A cohort study of Asian patients with NAFLD confirmed that, in the lean/normal-weight subgroup, sarcopenia and handgrip strength were significantly associated with ASCVD risk; muscle mass was negatively associated with ASCVD risk, while visceral adipose tissue was an independent predictor of advanced liver fibrosis (29). Therefore, our findings suggest that even in individuals with normal body weight, muscle-fat imbalance is a key risk factor for MASLD.

In marked contrast to the universal applicability of the FFM/FM ratio, WHR exhibited clear female specificity. Our data showed that WHR was an independent risk factor for MASLD only in females, and the combined FFM/FM + WHR model yielded a significantly higher AUC than FFM/FM alone, further supporting the unique predictive value of WHR in women. This finding has a clear physiological explanation: after menopause, declining estrogen levels shift fat distribution toward a “masculinized” pattern, favoring visceral fat accumulation. Mendelian randomization studies have confirmed a causal link between WHR and NAFLD risk (30, 31). Consequently, prevention and management of MASLD in women should adopt a “dual-pronged” strategy: increasing the FFM/FM ratio through muscle gain while reducing WHR through fat loss.

Beyond its hepatic implications, MASLD is increasingly recognized as a multisystem disorder closely intertwined with cardiovascular disease. MASLD is not only a liver disease but also a multisystem disorder closely associated with ASCVD (32). Notably, non-obese (lean) individuals with MASLD have an even higher risk of CVD than those with obese MASLD (33), a phenomenon partly attributed to sarcopenia and increased visceral adiposity. In this context, our finding that a lower FFM/FM ratio is independently associated with higher MASLD prevalence aligns with a recent study showing that the fat-to-muscle ratio (FMR) is an independent predictor of both liver fibrosis and ASCVD risk in Chinese patients with NAFLD (34). Although our cross-sectional design cannot establish causality and we did not directly assess cardiovascular endpoints, these converging lines of evidence support the potential value of the FFM/FM ratio as a marker for identifying individuals at risk for both hepatic and cardiovascular complications of MASLD.

When benchmarked against TyG and HSI, the combined WHR + FFM/FM model showed distinct sex-specific patterns (Schedule 5). In females, the combined model significantly outperformed TyG (AUC: 0.822 vs. 0.762, *p* = 0.046) and was comparable to HSI (*p* = 0.347), supporting the utility of BIA-derived body composition indices as a blood-free alternative to conventional metabolic markers in women. In males, the combined model was comparable to TyG (*p* = 0.923) but inferior to HSI (*p* = 0.002), likely because HSI incorporates liver enzymes and BMI, which are more directly linked to hepatic steatosis. In non-obese individuals, TyG and HSI showed higher AUCs than the combined model, suggesting that metabolic indices may have superior sensitivity in lean populations. Nevertheless, the practical advantages of BIA—non-invasive, radiation-free, and immediately available in routine check-ups—make it a valuable complementary tool for MASLD screening, particularly in females and in community settings where laboratory testing is less accessible. However, DCA did not demonstrate incremental net benefit when FFM/FM was added to WHR (Supplementary Figures 4 and 5), suggesting that its clinical utility in decision-making is limited.

Having established the robust associations of FFM/FM and WHR, we next examined the role of BMR, a metabolic indicator derived from body composition. BMR represents the energy expenditure required for basic bodily functions, accounting for 60–75% of total daily energy expenditure (35). Body composition plays an important role in maintaining BMR (36). Our study found that BMR was significantly positively associated with MASLD only in obese females, whereas the associations were inconsistent or non-significant in other subgroups. Several explanations may account for this selective association. First, obese individuals with MASLD typically have greater metabolically active fat mass, which directly increases resting energy expenditure. Second, chronic low-grade inflammation, commonly present in obesity-related MASLD, may drive a compensatory elevation in metabolic rate (37–39). Third, BMR in this study was derived from BIA-based FFM using the Cunningham formula rather than measured by indirect calorimetry, so measurement error or formula-specific bias cannot be entirely excluded. Given the lack of direct inflammatory markers (e.g., CRP, IL-6) in our data, these interpretations remain speculative and warrant further investigation. The limited nature of this association—being confined to obese females—contrasts with the robust and consistent associations observed for FFM/FM ratio and WHR across nearly all subgroups. This pattern suggests that BMR is primarily determined by FFM, and its association with MASLD is likely mediated through body composition rather than representing an independent metabolic driver. The lack of consistent BMR-MASLD associations across other subgroups further underscores that FFM/FM ratio and WHR—rather than BMR per se—are the key body composition indicators for MASLD risk stratification. Thus, while BMR was included as an exploratory variable, our findings do not support its routine use as a primary screening tool for MASLD in the general population.

Collectively, our findings suggest that BIA-derived body composition indices may be useful for epidemiological risk stratification and early risk detection, while their role in direct clinical decision-making appears limited. The lack of incremental DCA net benefit further indicates that WHR alone remains a strong clinical indicator.

Several limitations of this study warrant careful consideration when interpreting our findings. First, sample size and statistical power: although the total sample size was sufficient for multivariable regression analyses, the number of MASLD cases in the non-obese subgroup was small, resulting in limited statistical power for this subgroup. Larger studies are needed to confirm the findings in non-obese populations. Second, limitations of diagnostic and measurement methods: we used BIA to assess body composition, which correlates well with DXA and CT but cannot precisely distinguish subcutaneous from visceral fat. In addition, although abdominal ultrasound is widely accepted for screening hepatic steatosis in large populations, it has limited sensitivity for detecting mild steatosis (<20% hepatic fat content) (40), which may introduce misclassification bias. Any such misclassification would likely be non-differential and could attenuate the observed associations toward the null. Additionally, data on menopausal status were not available in this retrospective health check-up cohort. Given that the mean age of female MASLD cases was approximately 54 years, many were likely postmenopausal—a key determinant of body fat distribution and a potential confounder of the WHR-MASLD association in women. Future studies should collect menstrual history to enable adjustment for menopausal status when examining sex-specific associations in MASLD. Third, limitations for causal inference: the cross-sectional design precludes causal inference between the FFM/FM ratio and MASLD. Moreover, although we calculated E-values and performed sensitivity analyses, suggesting that our results are moderately robust to unmeasured confounders (e.g.physical activity, diet), residual confounding cannot be entirely excluded. Fourth, generalizability: the data were derived from a single hospital in southern China, and the generalizability of our findings requires validation in multi-center, geographically diverse populations. Fifth, lack of cardiovascular outcome data: this study focused on MASLD prevalence and did not collect outcome data on atherosclerosis or cardiovascular events, which limits direct evaluation of the cardiovascular predictive value of body composition indices. Future prospective studies incorporating cardiovascular outcome data are needed to validate the hypotheses raised in this study.

## Conclusion

In summary, the FFM/FM ratio shows a universal protective effect against MASLD, while WHR exhibits sex-specific predictive value only in women. The linear dose–response relationship between FFM/FM and MASLD underscores the need for continuous monitoring and optimization of muscle-fat balance, even in normal-weight individuals. For women, particularly postmenopausal women, WHR should be closely monitored. Moreover, low FFM/FM may serve as an early-warning marker for both MASLD and cardiovascular risk. BIA-derived body composition analysis may assist in identifying high-risk individuals for MASLD in large-scale health check-ups, supporting early risk detection and primary prevention.

## Data Availability

The raw data supporting the conclusions of this article will be made available by the authors, without undue reservation.

## Supplementary material

Supplementary Figure 1 Forest plot of core variables from Table 1.

Supplementary Figure 2 RCS plot for overall population.

Supplementary Figure 3 BMI‑stratified ROC curves for WHR, and combined FFM/FM model in predicting MASLD.

Supplementary Figure 4 Overall population DCA.

Supplementary Figure 5 Sex-stratified DCA.

## Glossary

BMI: body mass index
HBP: high blood pressure
SBP: systolic blood pressure
DBP: diastolic blood pressure
HR: heart rate
WHR: Waist-to-hip ratio
BMR: basal metabolic rate
FFM: fat-free mass
FM: fat mass
PBF: percent body fat
FFMI: fat-free mass index
FMI: fat mass index
FFM/FM: fat-free mass to fat mass ratio
ALT: alanine aminotransferase
AST: aspartate aminotransferase
GGT: gamma-glutamyl transferase
BUN: blood urea nitrogen
Cr: creatinine
UA: uric acid
FBG: fasting blood glucose
TC: total cholesterol
TG: triglyceride
LDL: low-density lipoprotein
HDL: high-density lipoprotein.
