# Supplementary material for: Association between body composition and metabolic dysfunction-associated steatotic liver disease in Chinese general population: a cross-sectional study stratified by sex and obesity status
Source: Front Public Health. 2026 Sep 17;14:1921631. doi: 10.3389/fpubh.2026.1921631 (PMC13627355; doi:10.3389/fpubh.2026.1921631)
Supplement: Supplementary file 1 [file Table_1.docx]

Schedule 1 | The comparison of clinical characteristics between participants with and without MASLD.

| Variables | Total(n=1209) | Non-MASLD(n=867) | MASLD(n=342) | *P*-value |
| --- | --- | --- | --- | --- |
| Male | 562(46.48%) | 338(38.99%) | 224(65.50%) | <0.001 |
| Age（year） | 49.23±12.10 | 49.02±12.09 | 49.77±12.15 | 0.333 |
| BMI (kg/m2) | 24.50±3.55 | 23.38±2.99 | 27.31±3.28 | <0.001 |
| HBP | 277(22.91%) | 170(20.17%) | 107(31.29%) | <0.001 |
| SBP (mmHg) | 125.40±19.47 | 123.21±19.45 | 130.80±19.18 | <0.001 |
| DBP (mmHg) | 74.17±12.52 | 72.41±12.22 | 78.52±12.65 | <0.001 |
| HR (beats/min) | 77.46±10.76 | 76.96±10.91 | 78.70±10.81 | 0.012 |
| WHR | 0.91±0.06 | 0.89±0.05 | 0.95±0.05 | <0.001 |
| BMR (kcal) | 1334.71±197.60 | 1290.30±179.83 | 1447.29±196.14 | <0.001 |
| FFM (kg) | 44.70±9.01 | 42.66±8.12 | 49.87±9.08 | <0.001 |
| FM (kg) | 19.38±6.26 | 17.56±5.11 | 23.97±6.56 | <0.001 |
| BFP (%) | 30.05±6.76 | 29.1±6.68 | 32.47±6.36 | <0.001 |
| FFMI (kg/m^2^) | 16.98±2.09 | 16.45±1.92 | 18.32±1.91 | <0.001 |
| FMI (kg/m^2^) | 7.47±2.46 | 6.89±2.16 | 8.94±2.56 | <0.001 |
| FFM/FM | 2.34 (1.89-2.91) | 2.44 (1.96-3.06) | 2.12 (1.75-2.61) | <0.001 |
| ALT(U/L) | 17 (12-25) | 15 (11-21) | 25 (18-38) | <0.001 |
| AST(U/L) | 20 (17-24) | 19 (16-22.5) | 22 (18-27) | <0.001 |
| GGT (U/L) | 23 (16-41) | 19 (14-32) | 39 (24-63) | <0.001 |
| BUN (mmol/L) | 4.77±1.25 | 4.72±1.27 | 4.89±1.21 | 0.037 |
| Cr (μmol/L) | 68 (59-82) | 65 (57-80) | 75 (63.25-86) | <0.001 |
| UA (μmol/L) | 343.94±91.69 | 322.22±81.21 | 398.99±93.78 | <0.001 |
| FBG (mmol/L) | 5.02 (4.65-5.41) | 4.97 (4.60-5.30) | 5.22 (4.80-5.76) | <0.001 |
| TC (mmol/L) | 4.96 (4.39-5.55) | 4.89 (4.33-5.46) | 5.19 (4.57-5.76) | <0.001 |
| TG (mmol/L) | 1.39 (0.94-2.04) | 1.17 (0.86-1.73) | 2.04 (1.53-2.83) | <0.001 |
| LDL (mmol/L) | 3.04±0.81 | 2.91±0.77 | 3.35±0.86 | <0.001 |
| HDL (mmol/L) | 1.34±0.35 | 1.41±0.34 | 1.16±0.31 | <0.001 |

Normally distributed variables are presented as mean ± SD and skewed variables as median (P25-P75); group comparisons were performed using the t‑test or Wilcoxon rank‑sum test as appropriate. Categorical variables are presented as frequencies (percentages) and compared using the χ² test. Abbreviations: BMI: body mass index; HBP: high blood pressure; SBP: systolic blood pressure; DBP: diastolic blood pressure; HR: heart rate; WHR: Waist-to-hip ratio; BMR: basal metabolic rate; FFM: fat-free mass; FM: fat mass; PBF: percent body fat; FFMI: fat-free mass index; FMI: fat mass index; FFM/FM: fat-free mass to fat mass ratio; ALT: alanine aminotransferase; AST: aspartate aminotransferase; GGT: gamma-glutamyl transferase; BUN: blood urea nitrogen; Cr: creatinine; UA: uric acid; FBG: fasting blood glucose; TC: total cholesterol; TG: triglyceride; LDL: low-density lipoprotein; HDL: high-density lipoprotein.

Schedule 2 | The comparison of clinical characteristics between male participants with and without MASLD stratified by BMI.

| Variables | | BMI<25kg/m^2^ | |  | BMI≥25kg/m^2^ | | *P*1 | *P*2 |
| --- | --- | --- | --- | --- | --- | --- | --- | --- |
|  |  | Non-MASLD（n=202） | MASLD（n=41） |  | MASLD（n=183） | |  |  |
| Age（year） | | 50.5±12.82 | 47.41±10.51 |  | 47.28±11.62 | | 0.15 | 0.945 |
| BMI (kg/m2) | | 22.37±1.88 | 23.59±1.31 |  | 28.40±2.62 | | <0.001 | <0.001 |
| HBP | | 42（20.79%） | 8（19.51%） |  | 59（32.24%） | | 0.853 | 0.108 |
| SBP (mmHg) | | 124.56±19.41 | 126.06±18.14 |  | 132.05±18.19 | | 0.650 | 0.058 |
| DBP (mmHg) | | 74.20±12.46 | 77.18±11.15 |  | 80.74±12.94 | | 0.158 | 0.104 |
| HR (beats/min) | | 75.04±10.69 | 75.27±10.41 |  | 79.08±10.96 | | 0.900 | 0.043 |
| WHR | | 0.89±0.05 | 0.92±0.04 |  | 0.97±0.05 | | <0.001 | <0.001 |
| BMR (kcal) | | 1413.56±111.23 | 1433.24±106.98 |  | 1581.92±130.16 | | 0.300 | <0.001 |
| FFM (kg) | 48.31±5.15 | | 49.23±4.96 |  | 56.10±6.02 | | 0.295 | <0.001 |
| FM (kg) | | 14.02±3.68 | 16.57±2.78 |  | 24.96±6.03 | | <0.001 | <0.001 |
| BFP (%) | | 22.31±4.84 | 25.16±3.40 |  | 30.69±4.91 | | <0.001 | <0.001 |
| FFMI (kg/m2) | | 17.30±1.13 | 17.60±0.89 |  | 19.60±1.34 | | 0.112 | <0.001 |
| FMI (kg/m2) | | 5.05±1.37 | 5.95±1.00 |  | 8.74±2.04 | | <0.001 | <0.001 |
| FFM/FM | | 3.44 (2.88-4.23) | 2.86 (2.70-3.31) |  | 2.31 (1.98-2.64) | | 0.001 | <0.001 |
| ALT(U/L) | | 17.50 (13-23) | 25 (20-40) |  | 30 (22-48) | | 0.006 | 0.219 |
| AST(U/L) | | 21 (18-25) | 22 (18-25) |  | 23 (19-29) | | 0.258 | 0.543 |
| GGT (U/L) | | 27 (17-45) | 45 (29-72) |  | 52 (35.50-72) | | 0.034 | 0.43 |
| BUN (mmol/L) | | 5.04±1.27 | 4.72±1.05 |  | 5.00±1.17 | | 0.138 | 0.157 |
| Cr (μmol/L) | | 81.10 (72-90) | 80 (71.20-89) |  | 82 (73-90) | | 0.647 | 0.853 |
| UA (μmol/L) | | 365.37±80.21 | 426.44±78.15 |  | 434.90±81.17 | | <0.001 | 0.544 |
| FBG (mmol/L) | | 4.97 (4.59-5.31) | 5.03 (4.77-5.28) |  | 5.29 (4.81-5.78) | | 0.003 | 0.24 |
| TC (mmol/L) | | 4.84 (4.29-5.42) | 5.18 (4.78-5.59) |  | 5.28 (4.70-5.86) | | 0.032 | 0.357 |
| TG (mmol/L) | | 1.38 (0.94-1.92) | 2.39 (1.74-3.47) |  | 2.26 (1.73-3.15) | <0.001 | | 0.521 |
| LDL (mmol/L) | | 2.99±0.74 | 3.25±0.79 |  | 3.49±0.87 | 0.042 | | 0.113 |
| HDL (mmol/L) | | 1.29±0.30 | 1.08±0.30 |  | 1.09±0.32 | <0.001 | | 0.918 |

Normally distributed variables are presented as mean ± SD and skewed variables as median (P25-P75); group comparisons were performed using the t‑test or Wilcoxon rank‑sum test as appropriate. Categorical variables are presented as frequencies (percentages) and compared using the χ² test. *P*1: MASLD group vs. NON- MASLD group among participants with BMI < 25 kg/m^2^; *P*2: BMI < 25 kg/m^2^ MASLD group versus BMI ≥ 25 kg/m^2^ MASLD group. Abbreviations: BMI: body mass index; HBP: high blood pressure; SBP: systolic blood pressure; DBP: diastolic blood pressure; HR: heart rate; WHR: Waist-to-hip ratio; BMR: basal metabolic rate; FFM: fat-free mass; FM: fat mass; PBF: percent body fat; FFMI: fat-free mass index; FMI: fat mass index; FFM/FM: fat-free mass to fat mass ratio; ALT: alanine aminotransferase; AST: aspartate aminotransferase; GGT: gamma-glutamyl transferase; BUN: blood urea nitrogen; Cr: creatinine; UA: uric acid; FBG: fasting blood glucose; TC: total cholesterol; TG: triglyceride; LDL: low-density lipoprotein; HDL: high-density lipoprotein.

Schedule 3 | The comparison of clinical characteristics between female participants with and without MASLD stratified by BMI.

| Variables | BMI<25kg/m^2^ | |  | BMI≥25kg/m^2^ | *P1* | *P2* |
| --- | --- | --- | --- | --- | --- | --- |
|  | Non-MASLD(n=409) | MASLD(n=34) |  | MASLD(n=84) |  |  |
| Age（year） | 47.25±11.54 | 57.59±9.16 |  | 53.17±13.05 | <0.001 | 0.074 |
| BMI (kg/m2) | 21.68±2.02 | 23.38±1.25 |  | 28.35±3.29 | <0.001 | <0.001 |
| HBP | 62(15.16 %) | 11 ( 32.35%) |  | 29(34.52%) | 0.009 | 0.821 |
| SBP (mmHg) | 119.89±19.1 | 127.28±21.68 |  | 131.80±20.48 | 0.033 | 0.287 |
| DBP (mmHg) | 69.82±11.37 | 71.56±11.13 |  | 77.14±12.16 | 0.391 | 0.023 |
| HR (beats/min) | 78.04±10.49 | 78.72±10.72 |  | 79.53±10.58 | 0.718 | 0.707 |
| WHR | 0.87±0.04 | 0.91±0.04 |  | 0.95±0.04 | <0.001 | <0.001 |
| BMR (kcal) | 1165.22±93.25 | 1184.82±86.91 |  | 1267.06±105.97 | 0.237 | <0.001 |
| FFM (kg) | 36.93±3.58 | 37.72±4.02 |  | 41.53±4.91 | 0.221 | <0.001 |
| FM (kg) | 16.16±3.62 | 19.23±2.56 |  | 27.34±6.40 | <0.001 | <0.001 |
| BFP (%) | 30.20±4.85 | 33.79±3.74 |  | 39.39±4.45 | <0.001 | <0.001 |
| FFMI (kg/m2) | 15.04±1.06 | 15.44±0.93 |  | 17.05±1.22 | 0.034 | <0.001 |
| FMI (kg/m2) | 6.60±1.50 | 7.91±1.09 |  | 11.26±2.52 | <0.001 | <0.001 |
| FFM/FM | 2.27 (1.98-2.71) | 1.93 (1.77-2.19) |  | 1.55 (1.37-1.79) | <0.001 | <0.001 |
| ALT(U/L) | 13 (10-17) | 19.50 (16-24.75) |  | 20 (14.75-25) | 0.036 | 0.289 |
| AST(U/L) | 18 (15-21) | 21.50 (18.25-26) |  | 19 (17-22) | 0.019 | 0.150 |
| GGT (U/L) | 15 (12-21) | 20.50 (14.25-37.25) |  | 23 (19-36) | 0.07 | 0.700 |
| BUN (mmol/L) | 4.44±1.19 | 5.04±1.83 |  | 4.66±1.00 | 0.008 | 0.270 |
| Cr (μmol/L) | 59 (54-64) | 59.50 (56-66.50) |  | 61.50 (56-66.25) | 0.315 | 0.692 |
| UA (μmol/L) | 280.52±58.96 | 323.63±94.11 |  | 337.87±76.80 | 0.013 | 0.395 |
| FBG (mmol/L) | 4.90 (4.54-5.20) | 5.31 (4.88-6.19) |  | 5.20 (4.80-5.68) | 0.006 | 0.347 |
| TC (mmol/L) | 4.89 (4.33-5.39) | 4.98 (4.29-5.64) |  | 5.13 (4.32-5.60) | 0.645 | 0.543 |
| TG (mmol/L) | 0.99 (0.77-1.36) | 1.50 (1.01-1.96) |  | 1.74 (1.27-2.14) | 0.001 | 0.075 |
| LDL (mmol/L) | 2.78±0.74 | 3.08±0.81 |  | 3.19±0.83 | 0.027 | 0.493 |
| HDL (mmol/L) | 1.54±0.31 | 1.27±0.23 |  | 1.29±0.26 | <0.001 | 0.773 |

Normally distributed variables are presented as mean ± SD and skewed variables as median (P25-P75); group comparisons were performed using the t‑test or Wilcoxon rank‑sum test as appropriate. Categorical variables are presented as frequencies (percentages) and compared using the χ² test. *P*1: MASLD group vs. NON- MASLD group among participants with BMI < 25 kg/m^2^; *P*2: BMI < 25 kg/m^2^ MASLD group versus BMI ≥ 25 kg/m^2^ MASLD group. Abbreviations: BMI: body mass index; HBP: high blood pressure; SBP: systolic blood pressure; DBP: diastolic blood pressure; HR: heart rate; WHR: Waist-to-hip ratio; BMR: basal metabolic rate; FFM: fat-free mass; FM: fat mass; PBF: percent body fat; FFMI: fat-free mass index; FMI: fat mass index; FFM/FM: fat-free mass to fat mass ratio; ALT: alanine aminotransferase; AST: aspartate aminotransferase; GGT: gamma-glutamyl transferase; BUN: blood urea nitrogen; Cr: creatinine; UA: uric acid; FBG: fasting blood glucose; TC: total cholesterol; TG: triglyceride; LDL: low-density lipoprotein; HDL: high-density lipoprotein.

Schedule 4 | AUC comparison for predicting MASLD by obesity status

| obesity status | Predictor | AUC (95% CI) | *P* (vs. Combined) |
| --- | --- | --- | --- |
| BMI≥25kg/m^2^  (n=686) | Combined | 0.706 (0.662-0.750) | - |
|  | WHR | 0.703 (0.659-0.747) | 0.381 |
|  | FFM/FM | 0.481 (0.431-0.531) | <0.001 |
| BMI<25kg/m^2^  (n=523) | Combined | 0.740 (0.685-0.796) | - |
|  | WHR | 0.741 (0.686-0.796) | 0.845 |
|  | FFM/FM | 0.559 (0.492-0.627) | <0.001 |

*P* values from DeLong test for correlated ROC curves.

Schedule 5 | Comparison of predictive performance of the combined WHR+FFM/FM model with TyG index and HSI across subgroups

| Subgroup | TyG AUC (95% CI) | HSI AUC (95% CI) | Combined AUC (95% CI) | *P*1 | *P*2 |
| --- | --- | --- | --- | --- | --- |
| Overall  (n=1209) | 0.792 (0.768-0.816) | 0.850 (0.829-0.871) | 0.804 (0.781-0.827) | 0.477 | 0.001 |
| Male  (n=562) | 0.775 (0.736-0.814) | 0.837 (0.805-0.870) | 0.778 (0.740-0.816) | 0.923 | 0.002 |
| Female  (n=647) | 0.762 (0.715-0.809) | 0.840 (0.804-0.877) | 0.822 (0.783-0.861) | 0.046 | 0.347 |
| BMI≥25kg/m^2^  (n=686) | 0.722 (0.678-0.766) | 0.742 (0.700-0.784) | 0.706 (0.662-0.750) | 0.574 | 0.173 |
| BMI<25kg/m^2^  (n=523) | 0.803 (0.750-0.856) | 0.805 (0.753-0.857) | 0.740 (0.685-0.796) | 0.116 | 0.078 |

*P* values from DeLong test for correlated ROC curves. Combined model includes WHR and FFM/FM as predictors. TyG, triglyceride-glucose index; HSI, hepatic steatosis index. *P*1, combined model vs. TyG; *P*2, combined model vs. HSI.
